# Reactive oxygen species generation by bovine blood neutrophils with different *CXCR1* (*IL8RA)* genotype following Interleukin-8 incubation

**DOI:** 10.1186/s12917-015-0418-5

**Published:** 2015-05-06

**Authors:** Joren Verbeke, Xanthippe Boulougouris, Carolien Rogiers, Christian Burvenich, Luc Peelman, Bart De Spiegeleer, Sarne De Vliegher

**Affiliations:** M-team and Mastitis and Milk Quality Research Unit, Department of Reproduction, Obstetrics, and Herd Health, Faculty of Veterinary Medicine, Ghent University, Salisburylaan, 133 Merelbeke, Belgium; Department of Comparative Physiology and Biometrics, Faculty of Veterinary Medicine, Ghent University, Salisburylaan, 133 Merelbeke, Belgium; Animal Genetics Laboratory, Department of Nutrition, Genetics, and Ethology, Faculty of Veterinary Medicine, Ghent University, Heidestraat, 19 Merelbeke, Belgium; Laboratory of Drug Quality & Registration, Department of Pharmaceutical Analysis, Faculty of Pharmaceutical Sciences, Ghent University, Ottergemsesteenweg, 460 Ghent, Belgium

**Keywords:** Bovine Neutrophil, Reactive Oxygen Species Generation, *CXCR1* Polymorphism, Interleukin 8

## Abstract

**Background:**

Associations between polymorphisms in the bovine *CXCR1* gene, encoding the chemokine (C-X-C motif) receptor 1 (IL8RA), and neutrophil traits and mastitis have been described. In the present study, blood neutrophils were isolated from 20 early lactating heifers with different *CXCR1* genotype at position 735 or 980. The cells were incubated with different concentrations of recombinant bovine IL-8 (rbIL-8) for 2 or 6 h and stimulated with phorbol 12-myristate 13-acetate (PMA) or opsonized zymosan particles (OZP). Potential association between *CXCR1* genotype and production of reactive oxygen species (ROS) was studied.

**Results:**

Although on single nucleotide polymorphisms (SNPs) may potentially affect CXCR1 function, SNPs c.735C > G and c.980A > G showed no association with ROS production with or without incubation of rbIL-8. Neutrophils incubated with rbIL-8 for 2 or 6 h showed higher PMA- and lower OZP-induced ROS production compared to control without rbIL-8.

**Conclusions:**

In the present study no association could be detected between superoxide production by isolated bovine neutrophils during early lactation and *CXCR1* gene polymorphism. IL-8 showed to possess inhibitory effects on ROS generation in bovine neutrophils.

## Background

Intramammary infection induces a fast influx of blood neutrophils into the site of infection [1]. Activated neutrophils eliminate invading pathogens by phagocytosis and a diverse array of oxygen-dependent and oxygen-independent killing mechanisms. A powerful mechanism is the generation of reactive oxygen species (ROS) or superoxide [2]. It is widely accepted that neutrophils play a pivotal role in mammary gland immunity. Since 1990, an overwhelming amount of evidence has been generated of neutrophil dysfunction around parturition and early lactation with consequences on the defense of the mammary gland [3]. For example, although *E.coli* strains may influence the severity of infection, the primary determinant of severity is the physiological state of the cow. Severity of experimentally induced *E.coli* mastitis during early lactation was tightly correlated with the pre-infection capacity of isolated blood neutrophils to generate ROS after zymosan and phorbol ester stimulation [4,5] and their chemotactic response as well [6]. Interleukin 8 (IL-8), an important chemokine in the innate immune response of the mammary gland [3], enhances ROS generation [7], causes chemotaxis [8] and delays apoptosis [9] of isolated bovine blood neutrophils *in vitro*. Interleukin 8 priming of isolated human neutrophils for higher superoxide production was mediated through CXCR1 (IL8RA) and not through CXCR2 (IL8RB) [10,11].

Many polymorphisms have been detected in the coding region of the bovine *CXCR1* gene [12,13]. Single nucleotide polymorphism (SNP) c.735C > G (dbSNP ID: rs208795699) causes an amino acid change in the third intracellular loop (p.His245Glu) potentially affecting G-protein binding and signal transduction. Furthermore, c.735C > G was found to be in full linkage disequilibrium with SNPs c.37A > T (rs380621468), c.38 T > A (rs110296731) and c.68G > A (rs133273369) causing amino acid changes p.Ile13Tyr and p.Gly23Glu in the N-terminus of CXCR1 known to have an important role in the first steps of binding IL-8 [13,14]. Associations between SNP c.735C > G and neutrophil functionality have been studied: blood neutrophils with genotype c.735GG showed a higher intracellular calcium release when stimulated with IL-8 and an increased ROS generation in response to PMA compared to neutrophils with genotype c.735CC (reviewed in [15]). Single nucleotide polymorphisms c.980A > G (rs43323012) and c.995A > G (rs43323013) cause changes in the C-terminus (p.Lys327Arg and p.His332Arg) and might interfere with adaptin-2 binding and receptor internalization [12].

Previous research indicated an association between SNP c.980A > G and likelihood of intramammary infection by major pathogens in early lactating heifers [13]. In the present study we wanted to know if *CXCR1* gene polymorphism (SNPs c.735C > G and c.980A > G) could affect neutrophil functionality. Blood neutrophils, with different *CXCR1* genotype, were isolated from 20 heifers during early lactation. ROS production as detected by chemiluminescence was measured following IL-8 incubation and stimulation with either PMA or opsonized zymosan particles (OZP). Freshly calved heifers were sampled because neutrophil functionality is reduced during this period [16].

We report the results of an association study between *CXCR1*SNPs c.735C > G and c.980A > G and blood neutrophil ROS. Additionally, we discuss the unexpected effect rbIL-8 had on neutrophil ROS depending on the stimulatory agent.

## Methods

### Study design

The experiment has been approved by the ethical committee of the Faculty of Veterinary Medicine, Ghent University (EC2013/190). Twenty Holstein heifers with different *CXCR1* genotype were included from 5 different commercial dairy herds. Selected heifers were not siblings, had no history of diseases and all quarters were culture negative for major mastitis pathogens. Within 24 h after calving, neutrophils were isolated from blood and incubated with 0, 40 or 400 ng/ml recombinant bovine IL-8 (rbIL-8) for 2 and 6 h. Next, neutrophils were stimulated with PMA or OZP and ROS generation was measured by chemiluminescence. Finally associations between ROS generation and genotype, incubation time and rbIL-8 concentration were statistically analyzed. The sample size (n = 20) was based on previous research demonstrating significant differences in ROS generation between 10 early and 10 mid lactating cows [16]. The incubation times were determined in a preliminary experiment in which blood neutrophils from 2 early lactating heifers were incubated for 2, 4, 6 and 18 h with 0, 40 or 400 ng/ml rbIL-8. A differential count of the isolated cells was performed to estimate the % neutrophils. Viability of neutrophils was measured after isolation and after each incubation time by trypan blue exclusion.

### Bacteriological culture

As mastitis can affect functionality of blood neutrophils [17], aseptic quarter milk samples were collected at the time of blood sampling and bacteriologically cultured. Ten μL of each sample was spread on blood-esculin and MacConkey’s agar and incubated aerobically for 24–48 h at 37°C. Bacteriological culture was performed according to National Mastitis Council (NMC) guidelines [18]. Four heifers were culture-positive in five quarters for major pathogens and excluded from the analysis. *Staphylococcus aureus*, esculin-positive cocci and *Escherichia coli* were isolated from 2 quarters of 1 heifers, 1 quarter of 1 heifer and 2 quarters of 2 heifers, respectively.

### *CXCR1* genotype

To include heifers with common and rare *CXCR1* genotypes (e.g. c.980AA), a sufficient number of heifers were genotyped before calving. A blood sample was taken from 60 Holstein heifers belonging to 5 herds and having an expected calving date between January and June 2014. Genotype at SNPs c.735C > G and c.980A > G was determined using a fluorescent multiprobe PCR assay as previously described [19]. Efforts were made to include sufficient heifers with genotype c.980AA or c.980AG. Of the 20 heifers included in the final analysis, 7, 6 and 7 had genotype c.735CC, c.735CG and c.735GG, respectively. Three, 5 and 12 had genotype c.980AA, c.980AG and c.980GG, respectively.

### Reactive oxygen species assay

Seventy-five mL blood was collected from the coccygeal vein using 8 mL Vacutainer tubes (Becton Dickinson, Erembodegem, Belgium) containing 150 μL of EDTA as anticoagulant. Blood neutrophils were isolated within 1 h of collection by hypotonic lysis of red blood cells and Histopaque 1077/1119 gradient (Sigma-Aldrich, Bornem, Belgium) centrifugation according to Siemens et al. [20]. Cell concentration was measured in triplicate with a Bürker chamber.

Two hundred thousand blood neutrophils were suspended in 200 μL of 1 × Hank's balanced salt solution (HBSS; Gibco, Life technologies, Carlsbad, CA) supplemented with 0, 40 or 400 ng recombinant bovine IL-8 (rbIL-8; Kingfisher Biotech, Saint Paul, MN) per mL and incubated for 2 or 6 h at 37°C in 2 mL test tubes. Next, blood neutrophils were pelleted by centrifugation at 1000 × *g* for 5 min and resuspended in 120 μl 1 × HBSS. Luminol (0.30 mmol/L Sigma-Aldrich) and PMA (100 ng/mL; Sigma-Aldrich) or OZP (750 μg/mL) were added to a final volume of 200 μL. Zymosan A (Sigma-Aldrich) was opsonized by washing the pellet with 60 and 30 mL 1 × phosphate-buffered saline (PBS; Gibco) (centrifugation at 200 × *g* for 10 min) followed by 1 h incubation at 37°C in 5 mL 1 × PBS and 35 mL bovine serum and two additional washing steps with 30 mL 1 × PBS (centrifugation at 200 × *g* for 10 min). Bovine serum was collected from the coccygeal vein of a healthy Holstein cow using 8 mL gel and clot activator tubes (Vacutest Kima, Piove di Sacco, Italy). Reactions of blood neutrophils primed by rbIL-8 were performed in duplicate. Chemiluminescence was measured every 60 sec for 90 min with a luminometer (TriStar^2^ LB 942 Multidetection Microplate Reader, Berthold Technologies, Bad Wildbad, Germany) and expressed in relative light units (RLU). Area under the curve (AUC) values (in 10^6^ RLU * s) were calculated to analyze the total ROS generation whereas peak values (RLUmax; in 10^3^ RLU) and time of peak values (Tmax; in min) were saved in the dataset to study the kinetics of ROS generation [5].

### Statistical analysis

Different linear mixed regression models (PROC MIXED, SAS 9.4, SAS Institute Inc.) were fit for AUC, RLUmax and Tmax after stimulation with PMA or OZP (6 outcome variables) and for SNP c.735C > G or c.980A > G (12 models in total). Heifer was added as random effect to correct for clustering of multiple observations (6) per heifer (RANDOM statement). The models included heifers’ genotype at position of the SNP, incubation (2 or 6 h) and rbIL-8 (0, 40 or 400 ng/ml) as categorical fixed effects. All two-way interactions between fixed effects were tested but removed from the models because they were non-significant (*P* > 0.05).

## Results

### Preliminary experiment

**Figure 1:**
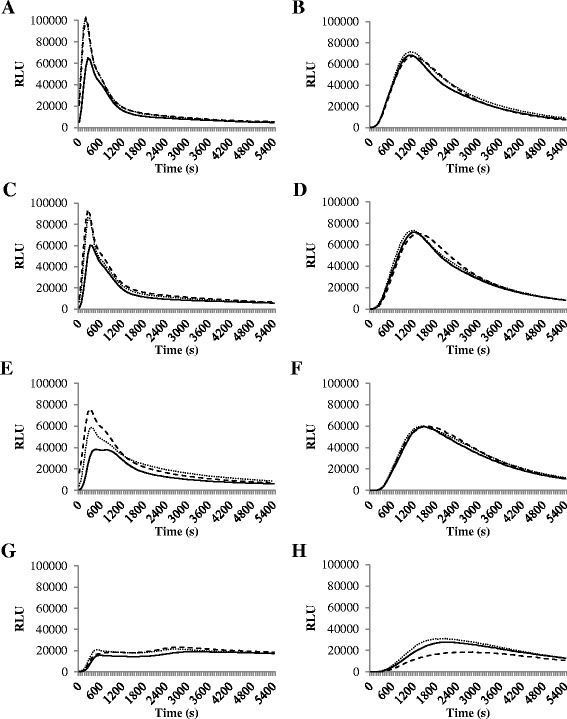
Effect of Interleukin-8 on reactive oxygen species generation of blood neutrophils.Preliminary experiment studying the reactive oxygen species (ROS) generation of blood neutrophils isolated from 2 early lactating dairy cows and incubated with 0 (**——**), 40 (••••) or 400 (− − −) ng recombinant bovine interleukin-8 (rbIL-8) per mL for 2 **(A** and **B)**, 4 **(C** and **D)**, 6 **(E** and **F)** or 18 **(G** and **H)** h measured using luminol chemiluminescence [expressed in relative light units (RLU)]. Prior to measurement, neutrophils were stimulated with phorbol 12-myristate 13-acetate **(A**, **C**, **E** and **G)** or opsonized zymosan particles **(B**, **D**, **F** and **H)**.

A differential count demonstrated 94.3% [standard deviation (SD) 0.1%] of the isolated cells to be neutrophils. The viability of the neutrophils was 100% immediately after isolation and decreased to 96% (SD 1%), 96% (SD 2%), 98% (SD 1%) and 81% (SD 3%) after 2, 4, 6 and 18 h isolation, respectively. Differences in viability between neutrophils incubated with 0, 40 or 400 ng/mL rbIL-8 were small (data not shown). Because of the low viability and strongly diminished ROS generation after 18 h incubation, neutrophils were incubated for 2 and 6 h (Figure 1). As expected [21], chemiluminescence increased fast after PMA stimulation with a clear peak and increased more gradually after OZP stimulation (Figure 1).

### Associations with ROS generation after PMA stimulation

Single nucleotide polymorphisms c.735C > G and c.980A > G were not associated with AUC, RLUmax or Tmax (*P* > 0.05) (Table 1). Incubation was associated with AUC, RLUmax and Tmax. Blood neutrophils incubated for 6 h showed higher AUC, RLUmax and Tmax values compared to blood neutrophils incubated for 2 h (*P* < 0.05). Concentration of rbIL-8 was associated with AUC and Tmax (*P* <0.01) and not with RLUmax (*P* = 0.17). Blood neutrophils incubated with 40 or 400 ng/mL showed higher AUC and Tmax values compared to blood neutrophils incubated without rbIL-8.

**Table 1:** **Statistical analysis of reactive oxygen species generation by blood neutrophils following phorbol 12-myristate 13-acetate stimulation**

**Polymorphism** ^**1**^	**Fixed effect**	**n** ^**2**^	**AUC** ^**3**^			**RLUmax** ^**4**^			**Tmax** ^**5**^		
			**β** ^**6**^	**SE** ^**7**^	***P*** ^**8**^	**β** ^**6**^	**SE** ^**7**^	***P*** ^**8**^	**β** ^**6**^	**SE** ^**7**^	***P*** ^**8**^
c.735C > G	Intercept		71	8		37	6		9.2	1.0	
	Genotype				0.69			0.94			0.47
	c.735CC	7	Ref.^9^	…		Ref.^9^	…		Ref.^9^	…	
	c.735CG	6	-6	11		-3	9		0.3	1.3	
	c.735GG	7	-9	11		-1	8		-1.2	1.3	
	Incubation				<0.01			<0.01			<0.05
	2 h		Ref.^9^	…		Ref.^9^	…		Ref.^9^	…	
	6 h		11	2		4	1		1.2	0.5	
	rbIL-8				<0.01			0.17			<0.01
	0 ng/ml		Ref.^9^	…		Ref.^9^	…		Ref.^9^	…	
	40 ng/ml		7	2		-3	2		3.1	0.6	
	400 ng/ml		5	2		-2	2		3.1	0.6	
c.980A > G	Intercept		66	11		29	9		8.9	1.5	
	Genotype				0.45			0.56			0.78
	c.980AA	3	Ref.^9^	…		Ref.^9^	…		Ref.^9^	…	
	c.980AG	5	10	14		12	11		0.7	1.8	
	c.980GG	12	-4	13		5	10		-0.2	1.6	
	Incubation				<0.01			<0.01			<0.05
	2 h		Ref.^9^	…		Ref.^9^	…		Ref.^9^	…	
	6 h		11	2		4	1		1.2	0.5	
	rbIL-8				<0.01			0.17			<0.01
	0 ng/ml		Ref.^9^	…		Ref.^9^	…		Ref.^9^	…	
	40 ng/ml		7	2		-3	2		3.1	0.6	
	400 ng/ml		5	2		-2	2		3.1	0.6	

^1^Linear mixed regression models describing the association between reactive oxygen species generation by blood neutrophils and *CXCR1* polymorphisms c.735C > G and c.980A > G, respectively. Neutrophils were incubated with 0, 40 or 400 ng recombinant bovine interleukin 8 per mL for 2 or 6 h and stimulated with phorbol 12-myristate 13-acetate,

^2^Number of heifers,

^3^Area under the curve values in 10^6^ RLU (relative light units) * s,

^4^Peak values in 10^3^ RLU.

^5^Time of peak values in min,

^6^Regression coefficient,

^7^Standard error,

^8^Overall *P*-value of the fixed effect,

^9^Reference,

All two-way interactions between the fixed effects were non-significant (P > 0.05) and removed from the model.

### Associations with ROS generation after OZP stimulation

Single nucleotide polymorphisms c.735C > G and c.980A > G were not associated with AUC, RLUmax or Tmax (*P* > 0.05) (Table 2). Incubation was associated with AUC and RLUmax and not with Tmax. Blood neutrophils incubated for 6 h showed higher AUC and RLUmax values compared to blood neutrophils incubated for 2 h (*P* < 0.05). Concentration of rbIL-8 was associated with AUC and RLUmax (*P* <0.01) and not with Tmax (*P* = 0.89). Blood neutrophils incubated with 40 or 400 ng/ml showed lower AUC and Tmax values compared to blood neutrophils incubated without rbIL-8. Differences were mainly in neutrophils incubated with rbIL-8 at a concentration of 400 ng/mL, AUC values were smaller.

**Table 2:** **Statistical analysis of reactive oxygen species generation by blood neutrophils following opsonized zymosan particles stimulation**

**Polymorphism** ^**1**^	**Fixed effect**	**n** ^**2**^	**AUC** ^**3**^			**RLUmax** ^**4**^			**Tmax** ^**5**^		
			**β** ^**6**^	**SE** ^**7**^	***P*** ^**8**^	**β** ^**6**^	**SE** ^**7**^	***P*** ^**8**^	**β** ^**6**^	**SE** ^**7**^	***P*** ^**8**^
c.735C > G	Intercept		253	32		69	10		31.7	4.0	
	Genotype				0.40			0.82			0.22
	c.735CC	7	Ref.^9^	…		Ref.^9^	…		Ref.^9^	…	
	c.735CG	6	-36	47		-6	14		-3.6	5.3	
	c.735GG	7	-63	45		-9	14		-9.2	5.1	
	Incubation				<0.01			<0.01			0.54
	2 h		Ref.^9^	…		Ref.^9^	…		Ref.^9^	…	
	6 h		20	7		5	2		1.2	1.9	
	rbIL-8				<0.01			<0.01			0.89
	0 ng/ml		Ref.^9^	…		Ref.^9^	…		Ref.^9^	…	
	40 ng/ml		-6	8		-3	2		-0.6	2.3	
	400 ng/ml		-26	8		-8	2		0.5	2.3	
c.980A > G	Intercept		248	48		63	15		32.9	6.0	
	Genotype				0.32			0.58			0.44
	c.980AA	3	Ref.^9^	…		Ref.^9^	…		Ref.^9^	…	
	c.980AG	5	13	61		12	18		-3.6	7.3	
	c.980GG	12	-51	54		-2	16		-7.8	6.4	
	Incubation				<0.01			<0.01			0.54
	2 h		Ref.^9^	…		Ref.^9^	…		Ref.^9^	…	
	6 h		20	7		5	2		1.2	1.9	
	rbIL-8				<0.01			0.17			0.89
	0 ng/ml		Ref.^9^	…		Ref.^9^	…		Ref.^9^	…	
	40 ng/ml		-6	8		-3	2		-0.6	2.3	
	400 ng/ml		-26	8		-8	2		0.5	2.3	

^1^Linear mixed regression models describing the association between reactive oxygen species generation by blood neutrophils and *CXCR1* polymorphisms c.735C > G and c.980A > G, respectively. Neutrophils were incubated with 0, 40 or 400 ng recombinant bovine interleukin 8 per mL for 2 or 6 h and stimulated with opsonized zymosan particles,

^2^Number of heifers,

^3^Area under the curve values in 10^6^ RLU (relative light units) * s,

^4^Peak values in 10^3^ RLU

^5^Time of peak values in min,

^6^Regression coefficient,

^7^Standard error,

^8^Overall *P*-value of the fixed effect,

^9^Reference,

All two-way interactions between the fixed effects were non-significant (P > 0.05) and removed from the model.

## Discussion

Research on genetic polymorphisms enlarges our knowledge on mammary gland immunity and helps us to understand why certain cows are more mastitis resistant than others [15]. Because of the important function of CXCR1 in the innate immunity of the mammary gland [8,22] and a quantitative trait locus for clinical mastitis in this region of the bovine genome [23], *CXCR1* polymorphisms form interesting study objects. In this study, an *in vitro* model was used to analyze the effect of *CXCR1* SNP on neutrophil functionality in a sample population of freshly calved heifers. Associations between *CXCR1* genotype and neutrophil ROS generation after rbIL-8 incubation and stimulation with PMA or OZP were studied in detail.

The association between SNP c.735C > G and PMA-induced ROS generation reported by Rambeaud et al. (2006) could not be confirmed in our model. In contrast to the previously demonstrated higher ROS generation [9], we observed numerically lower AUC values in c.735GG neutrophils compared to c.735CC neutrophils. Additionally, no significant interaction effects between c.735C > G and rbIL-8 concentration were observed. Based on previous research [13], we hypothesized a higher ROS generation and response to rbIL-8 in c.980AG compared to c.980GG neutrophils. However, neither c.980A > G nor the interaction between rbIL-8 concentration and c.980A > G were associated with AUC, RLUmax or Tmax of PMA- or OZP-induced ROS generation in our model. Despite potential effects on ligand binding, signal transduction and internalization of CXCR1 [12], no interaction between *CXCR1* SNPs and rbIL-8 incubation could be demonstrated. Human CXCR1 but not hCXCR2 was found to be important for activation of ROS [24]. Latter functional differences were partly attributed to amino acid sequence differences in the C-terminus causing a faster receptor phosphorylation and internalization of hCXCR2 compared to hCXCR1 [25-27]. In contrast to hCXCR1 and hCXCR2, the C-terminus of both bovine IL-8R show identical amino acid sequences [28]. Hence, activation of neutrophils could be mediated by both receptors and functional effects caused by *CXCR1* SNP might be compensated by a fully functional CXCR2, explaining similar rbIL-8 responses on ROS across *CXCR1* genotypes in our study.

Several studies demonstrated a priming effect of IL-8 on ROS generation in human neutrophils [10,29,30]. Enhancement of ROS generation already occurred after 5 min of IL-8 incubation [29] and was explained by intracellular calcium mobilization [30] and by activation of phospholipase D (PLD) [10], protein kinase C-ε (PKC-ε) [25] and phospholipase A_2_ [29]. To the best of our knowledge, Mitchell et al. [7] were the only to study the priming effect of IL-8 on bovine neutrophils. Intracellular ROS generation was measured by 2,7-Dichlorodihydrofluorecein diacetate (H2DCFDA) flow cytometry. Incubation with rbIL-8 for 18 h enhanced the *Mannheimia haemolytica*-induced ROS generation whereas incubation for 30 or 60 min had little effect [7]. In our study, ROS generation was measured using luminol chemiluminescence. This assay has the benefit that both intra- and extracellular ROS generation are measured and allows for measurement over time [31]. Neutrophils were exposed to the same concentrations of rbIL-8 as in the study of Mitchell et al. [7] but for only 2 or 6 h. We opted not to incubate for 18 h because our preliminary experiments showed a reduced viability and a strongly diminished ROS generation after such a long period. Associations were detected between rbIL-8 concentration and ROS generation indicating the presence of a functional IL-8 receptor on the isolated blood neutrophils. In contrast to research on human neutrophils [29,30], IL-8 also had inhibitory effects on neutrophils ROS generation in our model. Incubation with rbIL-8 had a positive effect on the total PMA-induced ROS generation but a negative effect on the total OZP-induced ROS generation. The stimulatory agent dependent effect could be explained by differences in the pathways of ROS generation by PMA and OZP [32]. Incubation with rbIL-8 might have simultaneously activated components of the pathway induced by PMA (e.g. PKC) while inhibiting components of the pathway induced by OZP (e.g. calcium mobilization).

## Conclusions

In conclusion, no differences in PMA- or OZP-induced ROS generation were detected in blood neutrophils isolated from early lactating heifers with different *CXCR1* c.735C > G and c.980A > G genotypes. The inhibitory effects of rbIL-8 on neutrophil ROS generation suggest a complex interaction between IL-8 and ROS generation in bovine neutrophils.

## Abbreviations

AUC: Area under the curve
CXCR1: Chemokine (C-X-C motif) receptor 1
IL-8: Interleukin 8
IL8RA: Interleukin 8 receptor A
NMC: National mastitis council
OZP: Opsonized zymosan particles
PMA: Phorbol 12-myristate 13-acetate
rbIL-8: Recombinant bovine interleukin 8
RLU: Relative light units
RLUmax: Peak value
ROS: Reactive oxygen species
SCC: Somatic cell count
SNP: Single nucleotide polymorphism
Tmax: Time of peak value
